# Supplementary material for: The interactome of intact mitochondria by cross-linking mass spectrometry provides evidence for coexisting respiratory supercomplexes
Source: Mol Cell Proteomics. 2017 Dec 8;17(2):216–32. doi: 10.1074/mcp.RA117.000470 (PMC5795388; doi:10.1074/mcp.RA117.000470)
Supplement: Supplemental Data [file supp_RA117.000470_133922_0_supp_23978_fzffwf.zip › spectra_annotation/mito_DR_spectra_annotation/158-1-1-1-18-1.pdf]

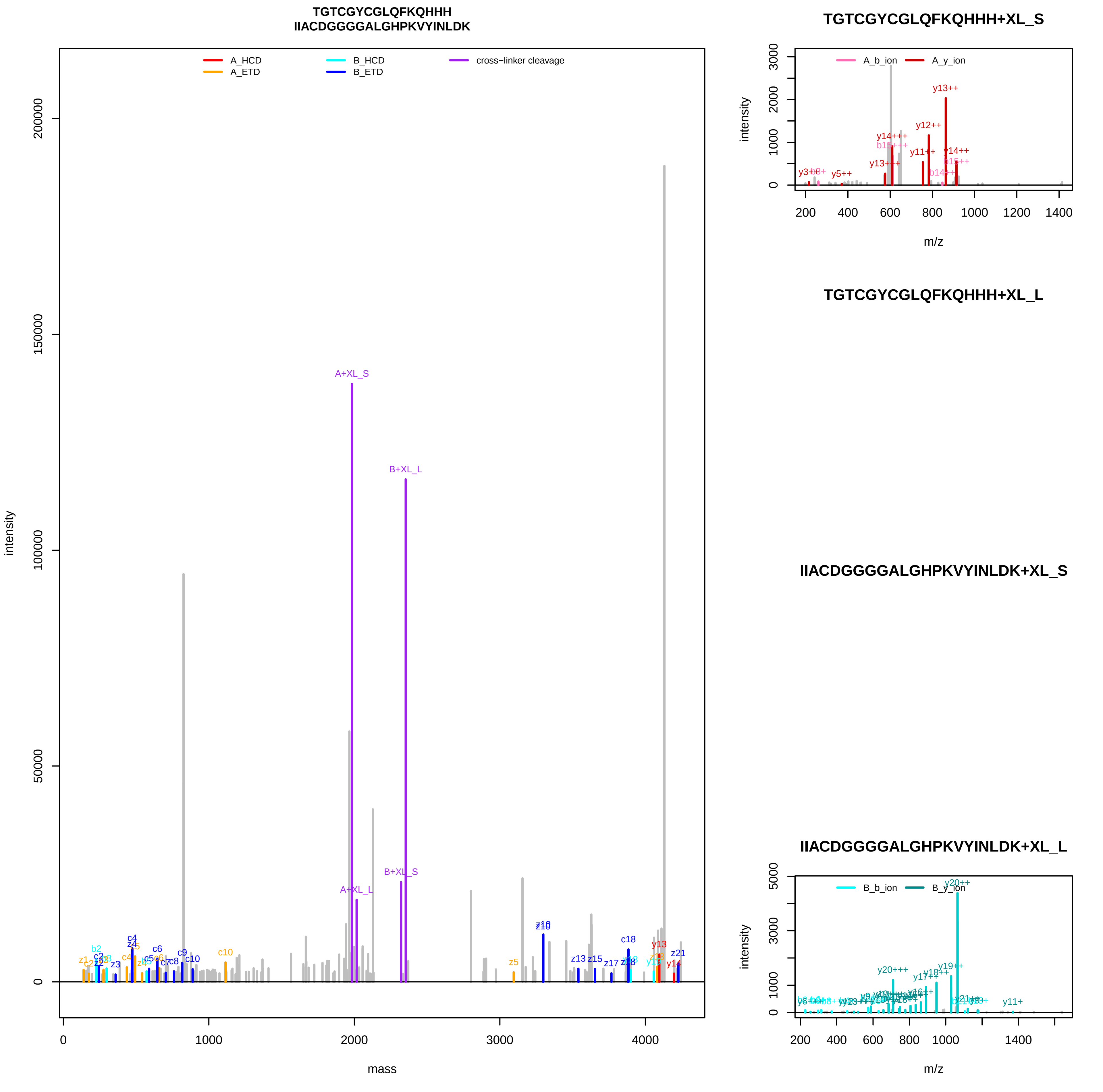
TGTCGYCGLQFKQHHH
IIACDGGGGALGHPKVYINLDK
A_HCD
A_ETD
B_HCD
B_ETD
cross−linker cleavage
A+XL_S
B+XL_L
B+XL_S
A+XL_L
intensity
mass
0
50000
100000
150000
200000
0
1000
2000
3000
4000
TGTCGYCGLQFKQHHH+XL_S
A_b_ion
A_y_ion
y13++
y12++
y14+++
y11++
y14++
y13+++
y5++
m/z
200
400
600
800
1000
1200
1400
3000
2000
1000
TGTCGYCGLQFKQHHH+XL_L
IIACDGGGGALGHPKVYINLDK+XL_S
IIACDGGGGALGHPKVYINLDK+XL_L
B_b_ion
B_y_ion
y20++
y20+++
y19++
y18++
y17++
y11+
5000
3000
1000
0
